# Illegal Cremation

**Published:** 1909-07-03

**Authors:** 


### Medico-Legal Points.

### ILLEGAL CREMATION.

At the Lambeth Police Court early in this year a case was heard which has some instructive applications. A woman was summoned for carrying out, procuring, and taking part in the burning of the remains of a child, otherwise than in accordance with the regulations and provisions of the Cremation Act, 1902. An unmarried woman stated that on December 29 she gave birth to a still-born child. She wrapped the body up and gave it to a friend to take to the defendant. The friend carried the body and half a sovereign to the defendant who said that the only thing to be done was to take the body to an undertaker's. They did so, and the defendant asked the undertaker to have the body buried. The undertaker replied that he could not possibly take it without a doctor's certificate. They returned to the defendant's house, and the witness left the body there. The defendant said she would get rid of it the same night, but she expressed much surprise that the dead child should have been sent to her. After the return from the undertaker's the defendant admitted having burned the body in her kitchen stove. The magistrate remarked that it seemed hard that the defendant should be made the scapegoat for the offences of others, but she took upon herself the responsibility of dealing with the body of a child, of the history of whose birth she knew nothing, and brought herself within the section of the Act. She would have to pay a penalty of £10 and 2s. costs within a fortnight.

The method of disposing of dead bodies by burning has always been much used in the East, but in Europe it seems to be opposed to the instincts of most people, and has never been generally adopted. There is, however, no doubt that, apart from sentimental considerations—which are to a large extent based on misconceptions and obstinate prejudices—cremation is, from a purely sanitary point of view, preferable to interment. Sanitary reformers have for some time advocated its adoption in this country, but the general opinion was for a very long time that it was illegal, and consequently cremations seldom or never took place.

A case, however, came before Mr. Justice Stephen, about the time when a strong and earnest advocacy of cremation was being conducted by a small band of reformers in the face of the most active and virulent opposition: this judge decided that, if conducted in such a way as not to offend public feeling, or prevent proper investigation being made as to the cause of death, cremation is not illegal (R. *v.* Price, 1883). Since that decision crematories have been started, and those who desire to set an example of disposing of the dead in such a manner as to prevent the danger of their poisoning the living can cremate them. In 1902 an Act was passed to legalise and regulate cremations.

Burial authorities are empowered to provide and maintain crematories, and the Secretary of State for Home Affairs is required to make regulations as to the maintenance and inspection of crematories, and to prescribe in what cases and under what conditions human cremation may take place. These were accordingly made in March, 1903.

By Section 8 every person, whether medical or lay, who shall contravene any of the regulations so made, or shall knowingly carry out or procure or take part in the burning of any human remains, except in accordance with the regulations and the provisions of the Act, shall (in addition to any liability or penalty which he may otherwise incur) be liable on summary conviction to a penalty not exceeding £50. Provided that any person aggrieved by any conviction may appeal therefrom to Quarter Sessions. Every person who wilfully makes any false declaration or representation, or signs or utters any false certificate, with a view to procuring the burning of any human remains, shall (in addition to any penalty or liability which he may otherwise incur) be liable to imprisonment with or without hard labour not exceeding two years. Every person who with intent to conceal the commission or impede the prosecution of any offence, procures or attempts to procure the cremation of any body, or with such intent makes any declaration or gives any certificate under this Act is liable to conviction on indictment to penal servitude for a term not exceeding five years. Under the regulations of the Home Secretary no cremation may take place except in a crematorium approved by him, nor until the death has been registered by two independent medical practitioners, and the written authority of the medical referee has been obtained. This must be withheld if any suspicious circumstances come to his knowledge.

